# The combined effect of magnetic and electric fields using on/off infrared light on the blood sugar level and the diameter of Langerhans islets of diabetic mice

**DOI:** 10.14202/vetworld.2020.2286-2293

**Published:** 2020-10-31

**Authors:** S. Suhariningsih, Suryani Dyah Astuti, Saikhu Akhmad Husen, Dwi Winarni, Dian Astri Rahmawati, Akhmad Taufiq Mukti, Alfian Pramudita Putra, Muhammad Miftahussurur

**Affiliations:** 1 Department of Physics, Faculty of Sciences and Technology, Universitas Airlangga, Surabaya 60115, Indonesia; 2 Biophysics and Medical Physics Research Group, Faculty of Sciences and Technology, Universitas Airlangga, Surabaya 60115, Indonesia; 3 Department of Biology, Faculty of Science and Technology, Universitas Airlangga, Surabaya, 60115, Indonesia; 4 Department of Fish Health Management and Aquaculture, Faculty of Fisheries and Marine, Universitas Airlangga, Surabaya 60115, Indonesia; 5 Biomedical Engineering Study Program, Department of Physics, Faculty of Sciences and Technology, Universitas Airlangga, Surabaya 60115, Indonesia; 6 Biomedical Signals and Systems Research Group, Faculty of Sciences and Technology, Universitas Airlangga, Surabaya 60115, Indonesia; 7 Department of Internal Medicine, Division of Gastroentero-Hepatology, Faculty of Medicine, Institute of Tropical Diseases, Universitas Airlangga, Surabaya, Indonesia

**Keywords:** blood sugar level, diabetic mice, electric field, infrared light, Langerhans islets diameter, magnetic field

## Abstract

**Background and Aim:**

At present, diabetes is treated with oral antidiabetic medicines, such as sulfonylureas and thiazolidine, as well as insulin injection. However, these methods have several shortcomings. Therefore, alternatives for treating diabetes mellitus (DM) are needed. This study aims to determine the combined effect of magnetic and electric fields on blood sugar levels and the diameter of Langerhans islets of diabetic mice.

**Materials and Methods:**

Induction of DM in mice was carried out by administering lard for 2 weeks and continued with an intraperitoneal injection of streptozotocin, dissolved in a 4.5 pH citrate buffer, and administered in a dose of 30 mg/kg bodyweight for 5 days. Treatments were used in combination with magnetic and electric fields using on/off infrared light. Blood samples were pipetted through the tip of mice’s tails to establish the blood sugar level for each individual mouse. Histology preparation of the pancreas organ was affected using the histology standard as well as hematoxylin and eosin staining methods. Langerhans islet diameter data were analyzed using analysis of variance followed by Duncan’s multiple range test. Data analysis was performed at ssssssss=0.05.

**Results:**

The results showed that the combined treatment of permanent magnetic and unidirectional electric fields (PS) caused changes in blood sugar levels that were not significantly different from the normal control group. The PS treatment improved the diameter of the Langerhans islets but not to a significant degree compared to other treatments.

**Conclusion:**

The use of PS treatment is effective for reducing the blood sugar levels of diabetic mice and improving the diameter of their Langerhans islets.

## Introduction

Diabetes is typically treated with oral ­antidiabetic medicines, such as sulfonylureas and thiazolidine, as well as insulin injection [1,2]. However, these methods can cause hypoglycemia [3], weight gain [4], and digestive tract disorders [5]. Accordingly, alternatives for treating diabetes mellitus (DM) are needed. One such alternative involves combining a magnetic field, electric field, and infrared light [6]. Based on the results of these studies, the current paper ­hypothesizes that the combination of electric fields, magnetic fields, and infrared rays can reduce the diameter of the Langerhans islets of diabetic mice (*Mus musculus*). Electric fields can have positive physical, cellular, and physiological effects on animals included in experiments. An electric field can induce changes in transmembrane potential and have a dilating effect on blood vessels by stimulating the release of endothelial-derived nitric oxide, which can give rise to the relaxation of smooth muscle in blood vessel walls [1]. The dilation of blood vessels can increase the blood flow rate, which, in turn, will lead to a decrease in blood viscosity [7,8]. Similar to electric fields, magnetic fields can also lower blood viscosity [7]. A magnetic field can control the conformational arrangement of aggregated erythrocytes, making it easier to follow the dynamics of blood flow in the vessels. Erythrocyte aggregate conformation regulation is possible due to the presence of hemoglobin containing ferrous ions, which imbues red blood cells/erythrocytes with paramagnetic properties [9] and enables them to be influenced by external magnetic fields (permanent or electromagnetic).

It is known that the body contains electrolyte fluid, a solution of ions (positively and negatively electrically charged) with the same vibrational frequency as infrared. Accordingly, when the body is exposed to infrared rays, it will absorb photons/infrared energy, resulting in electrons being excited, before transitioning to a ground state by emitting biophotons, which generate heat and will increase body temperature or give rise to hyperthermia. Hyperthermic conditions will increase the concentration of free Calcium (Ca^2+^) in the cytosol, which can eventually induce translocation of glucose transporter 4 (GLUT4) in striated muscle cells; this will increase glucose uptake from the blood [5], thereby increasing the blood flow rate through vasodilation effects [10,11]. As such, an increase in temperature in the red and infrared spectrum can be used for additional treatment in medicine with a relatively fast healing time [12].

Hyperglycemia occurs when glucose levels in the blood increase. This condition is a primary characteristic of DM. Hyperglycemia conditions can result in increased levels of reactive oxygen species and reactive nitrogen species, which are highly reactive molecules that can cause cellular damage and oxidative stress [4]. Increased blood glucose levels can lead to increased blood viscosity. An increase in blood viscosity will cause a decrease in blood flow rate, which can cause a decrease in the amount of insulin that can bind to its receptors in the tissue and is associated with insulin resistance [6]. A decrease in excitability and insulin sensitivity causes the amount of GLUT4 translocated to the cell surface to decrease so that the concentration of glucose taken from the blood is also reduced; as a result, blood glucose levels will remain high [5]. High blood glucose levels can cause β-cell dysfunction in the Langerhans islets of the pancreas because they will continue being stimulated to secrete insulin, which can cause a decrease in the diameter of Langerhans islets in the pancreas [13].

To date, DM treatment has been carried out using oral antidiabetic drugs, such as sulfonylurea and thiazolidine, as well as insulin injection. Both of these methods present several disadvantages, for example, hypoglycemia, weight gain, and gastrointestinal disorders [4]. Therefore, alternatives to drugs and insulin injection are needed for the treatment of DM. One such treatment involves combining magnetic fields, electric fields, and infrared rays.

The current research objective was to determine the effect of this combined treatment on reducing blood sugar levels and the diameter of Langerhans islets in diabetic mice. Accordingly, this study hypothesizes that a combination of electric, magnetic, and infrared fields can reduce blood sugar levels and the diameter of Langerhans islets in diabetic mice.

## Materials and Methods

### Ethical approval

This study was approved by the Ethical Committee of Faculty of Veterinary in Universitas Airlangga, Surabaya, Indonesia, with the reference number 2.KE.055.01.2018.

### Study period and location

This study was conducted at Veterinary and Histology Laboratory of Biology Department, Biophysic Laboratory of Physic Department, Faculty of Science and Technology, Universitas Airlangga, from June to October 2016 .

### Samples

This study included 30 BALB/c strain adult male mice. The sample was split into two groups, those were, a healthy group (3 mice) and a diabetic group (27 mice). The diabetic group was induced using lard as an oral high-fat diet before induced using streptozotocin (STZ). The dose of lard was given at 0.3 mL/30 g bodyweight (BW) every 2 days for 20 days. The induction procedure was affected based on Novelli *et al*. [14], that is, mice were induced with STZ dissolved in a pH 4.5 citrate buffer at a dose of 30 mg/kg BW intraperitoneally for 5 consecutive days. On the 7^th^ and 14^th^ days after induction, the blood sugar levels of mice were evaluated. Mice with a sugar level higher than 200 mg/dL were categorized as diabetic mice. The diabetic mice were grouped into nine subgroups, that is, one control group and eight treatment groups, with three mice in each group, based on the Federer formula for sample size.

#### Apparatus chamber for the treatment of samples

A solenoid with a diameter of 1 mm and a length of 12 cm was used as an alternating magnetic field source. The alternating magnetic field produced by the solenoid ranged from 500 to 900 μT. Conversely, neodymium was used as a permanent magnetic field source. It was arranged as 42 pieces of serial wire. The produced permanent magnetic field ranged from 100 to 125 mT. Contrastingly, an alternating (electromagnetic) magnetic field was created using an electromagnetic coil fed by an alternating current so that the emitted magnetic induction was an alternating magnetic field ranging from 500 to 900 μT. In this study, two different types of electrical fields were used, that is, static and dynamic. The alternating electrical field was derived from an alternating current voltage source of 155 V with a frequency of 16 kHz. The distance between the two plates was 30 cm. Thus, the static electrical field was 900 V/30 cm. A near-infrared (NIR) light with a wavelength of 941 nm had an intensity of 4 mW/cm^2^, which was generated by a light-emitting diode (LED) with 1 mW output at a 20 cm range. The generator for all sources was placed inside the apparatus chamber and the mice were put inside a plastic container that was exposed to all sources used.

#### Treatment samples

**Figure-1:**
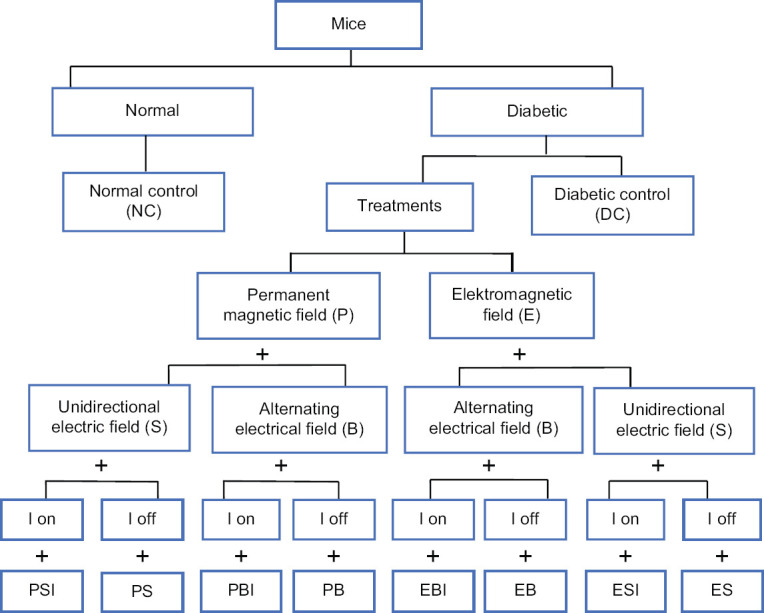
Set up of treatment samples, permanent magnetic and the unidirectional electric fields in infrared on (PSI); permanent magnetic and the unidirectional electric fields in infrared off (PS); permanent magnetic and an alternating electrical fields in infrared on (PBI); permanent magnetic and an alternating electrical fields in infrared off (PB); electromagnetic and an alternating electrical fields in infrared on (EBI); electromagnetic and an alternating electrical fields in infrared off (EB); electromagnetic and the unidirectional electric fields in infrared on (ESI); and electromagnetic and the unidirectional electric fields in infrared off (ES).

Each subgroup of mice was exposed to the magnetic and the electrical fields produced by the generators inside the apparatus chamber, as shown in Figure-1 (excluding the normal control [NC], and diabetic control [DC] groups). The treatments were varied as follows: (1) Permanent magnetic and unidirectional electric fields for infrared-on light (PSI); (2) permanent magnetic and unidirectional electric fields for infrared-off light (PS); (3) permanent magnetic and alternating electrical fields for infrared-on light (PBI); (4) permanent magnetic and alternating electrical fields for infrared-off light (PB); (5) electromagnetic and alternating electrical fields for infrared-on light (EBI); (6) electromagnetic and alternating electrical fields for infrared-off light (EB); (7) electromagnetic and unidirectional electric fields for infrared-on light (ESI); and (8) electromagnetic and unidirectional electric fields for infrared-off light (ES). Animals included in the experiment that were already diabetic were treated for 15 min every day for 30 days.

#### Diameter measurement of Langerhans islets

**Figure-2:**
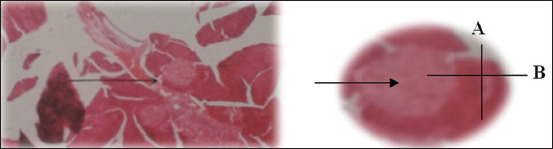
The diameter of Langerhans islets, (A+B)/2 (A is the shortest diameter and B is the longest diameter).

The diameter of Langerhans islets was measured on a serial histological section of the pancreas. The largest Langerhans islet diameter was used and was incorporated into the calculation results of additional data (Figure-2).

### Statistical analysis

Data were statistically analyzed using analysis of variance followed by Duncan’s multiple range test (DMRT) with α=0.05.

## Results

**Figure-3:**
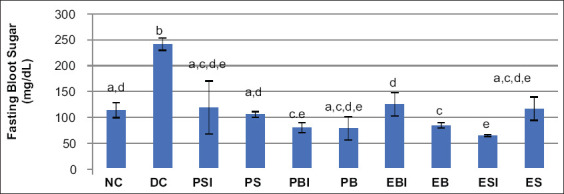
The fasting blood sugar level of all treatment groups. Different letters (a-e) above indicate that there are significant differences based on the t-test at α=0.05. normal control (NC), and diabetic control (DC), permanent magnetic and the unidirectional electric fields in infrared on (PSI); permanent magnetic and the unidirectional electric fields in infrared off (PS); permanent magnetic and an alternating electrical fields in infrared on (PBI), permanent magnetic and an alternating electrical fields in infrared off (PB); electromagnetic and an alternating electrical fields in infrared on (EBI); electromagnetic and an alternating electrical fields in infrared off (EB); electromagnetic and the unidirectional electric fields in infrared on (ESI); and electromagnetic and the unidirectional electric fields in infrared off (ES).

The combined effect of the magnetic and electric fields for on/off infrared light on the blood sugar levels of mice: The combined effect of the magnetic and electric fields in on/off infrared light on blood sugar level was obtained using the blood sugar level measured at the end of the treatment after the animals had fasted for 6 h. The t-test results for the data on fasting blood sugar levels in diabetic mice showed that the fasting blood sugar levels in the DC group were significantly different from other groups. Fasting blood sugar levels in the NC group differed significantly from the KD, PBI, EB, and ESI groups. However, fasting blood sugar levels in the KN group were not significantly different from those in the PSI, PS, PB, EBI, and ES groups. The highest fasting blood sugar levels were found in the DC group. Meanwhile, the lowest fasting blood sugar levels were found in the ESI group. A diagram for the results of fasting blood sugar levels in all treatment groups using the t-test is presented in Figure-3.

**Figure-4:**
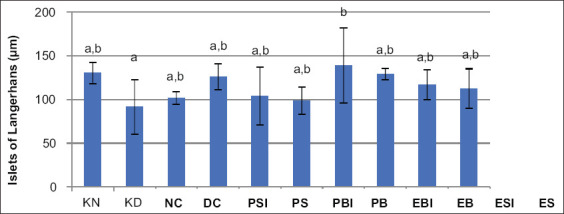
The graph of Langerhans islets diameter of all treatment groups. Different letters (a and b) above show significant differences based on DMRT at α=0.05. Normal control (NC), and diabetic control (DC), permanent magnetic and the unidirectional electric fields in infrared on (PSI); permanent magnetic and the unidirectional electric fields in infrared off (PS); permanent magnetic and an alternating electrical fields in infrared on (PBI); permanent magnetic and an alternating electrical fields in infrared off (PB); electromagnetic and an alternating electrical fields in infrared on (EBI); electromagnetic and an alternating electrical fields in infrared off (EB); electromagnetic and the unidirectional electric fields in infrared on (ESI); and electromagnetic and the unidirectional electric fields in infrared off (ES).

The combined effect of the magnetic and electric fields in on/off infrared light on the diameter of Langerhans islets: The combined effect of the magnetic and electric fields in on/off infrared light on the diameter of Langerhans islets was derived from the diameter measurement results. The effect of combining magnetic fields, electric fields, and infrared rays on the diameter of Langerhans islets was derived from the results of measuring the diameter of Langerhans islets. Based on the results of the Duncan test, the diameter of Langerhans pancreatic islets in diabetic mice showed no significant difference between the DC and NC groups. The Langerhans islet diameter of the DC group was not significantly different from the PSI, PS, PBI, PB, EB, ESI, and ES groups. However, the diameter of the Langerhans islets in the DC group differed significantly from that of the EBI group. The diameter was measured using the DMRT in all treatment groups, as shown in Figure-4.

**Figure-5:**
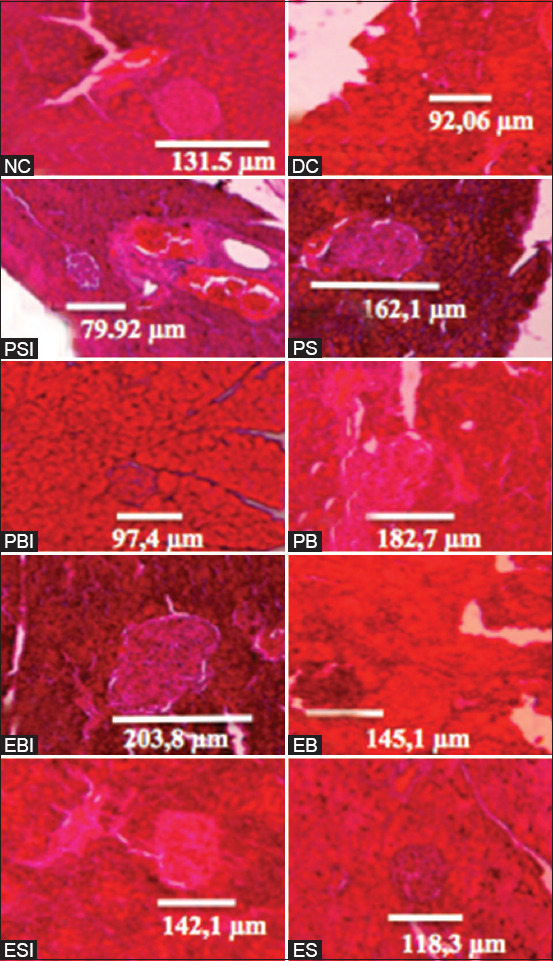
Histological incision of Langerhans islets in the pancreatic tissue by HE staining for all treatment groups; NC, DC, PSI, PS, PBI, PB, EBI, EB, ESI, and ES (Bar scale=100 μm).

An image of the diameter measurement results of the Langerhans islets in all treatment groups is presented in Figure-5.

## Discussion

In the present study, the measurements of fasting blood sugar levels in the diabetic group showed a hyperglycemic condition. These results indicate that the induction of DM through a high-fat diet and peritoneal injection of STZ resulted in hyperglycemia in mice, causing the blood sugar levels of mice to rise abnormally. The target cell receptor that receives insulin will activate tyrosine kinase, which will cause glycogen synthesis activity in the liver, thereby increasing glucose transport into peripheral tissue [15].

Damage to pancreatic β cells may result in an inability to produce normal amounts of insulin; subsequently, a degree of damage will affect the size of Langerhans islets. The Langerhans islets vary in size according to the number of their constituent cells. These islets are responsible for insulin secretion by pancreatic β cells. Under normal circumstances, insulin secretion will not be disturbed by the presence of diabetic agents [16]. Histologically, the diameter of Langerhans islets in mice has a diameter approximately 2 times greater (116±80 μm) than human islets (50±29 μm) [17]. Pancreatic islets will experience a change in diameter under the condition of hyperglycemia. This was observed in the islet diameter of the DC group in this study, which had an average diameter of 91.7 μm. This indicated a significant decrease when compared to the NC group, which had an average diameter of 130.2 μm.

Various treatments using a combination of PSI, PS, PBI, PB, EBI, EB, ESI, and ES for 15 min daily for 30 days (Figure-3) show that combining magnetic and electric fields in on/off infrared light can suppress an increase in the fasting blood sugar levels of ­diabetic mice. In addition, no significant difference was observed between the fasting blood sugar levels of the DC and NC groups compared to the NC group. The reduction in the blood sugar level when using these treatment approaches was significant compared to the NC group, to the degree that it is advised not to be used for reducing hyperglycemia in diabetic individuals.

The electric dipole moment can suppress an increase in fasting blood sugar levels in diabetic mice by influencing electrolytes in the blood. Negatively charged electrolytes will tend to move in the opposite direction of the applied electric field. Contrastingly, positively charged electrolytes will move in the direction of a given external electric field.

If there is no external magnetic field, the magnetic dipole will adopt a random orientation (Figure-4a); when a magnetic field is applied, the hemoglobin molecules will tend to align their position in accordance with the direction of the applied magnetic field. In addition, the aligned position of hemoglobin due to the magnetic field can improve oxidation in damaged body cells because oxygen molecules, when bound by iron ions, will be in a symmetrical position, which will facilitate its easy removal.

Results shown in Figure-3 indicate that the fasting blood sugar of the DC group was significantly different from the NC group. The diameter of Langerhans islets in the DC group did not differ from the PSI, PS, PBI, PB, EB, ESI, and ES groups. This means that all treatments were significant in terms of improving the functioning of Langerhans islets because their diameters closely matched that observed for the normal group. Meanwhile, the diameter of Langerhans islets in the DC group was significantly different from the EBI group. However, the diameter increase in the EBI treatment group was significant when compared to the NC group and if the condition continues, it could cause hyperinsulinemia.

The optimal treatment for improving the blood sugar levels of diabetic mice in this study was established as a combination of permanent magnetic field therapy devices. The use of PS supports the research findings of Gmitrov [7]. The combined treatment of permanent magnetic and unidirectional electric fields (PS) can restore the blood sugar levels of diabetic mice close to blood sugar levels of NC group. Likewise, the diameter of Langerhans islets after treatment with the PS approach was very similar to the NC group. Although the EB combination also significantly increased islet diameter, it did not significantly improve blood sugar levels.

Changes in blood sugar levels and the diameter of Langerhans islets are the primary determinants of the functional regulation and beta-cell mass of Langerhans islets. However, chronic hyperglycemia will result in glucotoxicity in the beta cells; glucotoxicity will cause dysfunction and changes in the beta-cell mass, leading to a decrease in insulin secretion. Abnormalities in the mechanism of insulin secretion will reduce glucose intake by cells while rising blood sugar levels will result in a state of hyperglycemia. In agreement with previous study [1] using the same treatments, showed increased insulin sensitivity, decreased blood glucose levels, and the need for using a homeostatic model assessment of insulin resistance (HOMA-IR). In all treatment combinations in the current study, fasting blood glucose decreased significantly compared to the DC group, while insulin levels did not show any significant differences. The changes in both these parameters due to treatment also caused the HOMA-IR index to decrease. All treatment groups showed a significant reduction in HOMA-IR compared to the DC group. When insulin sensitivity was restored, higher levels of blood glucose could be absorbed into the skeletal muscle cells, thereby lowering blood glucose levels and increase the diameter of Langerhans [1]. Significant changes in the histological structure of Langerhans islets are typical pathological features that are often found in DM patients and animal models [18].

A static magnetic field does not change due to a permanent magnet; contrastingly, an electromagnetic field is produced by the movement of charged particles. The magnetic field generates interactions between the two poles, producing attraction, repulsion, or circular motion between them. Rotational movements occur because one pole receives attraction and repulsion from another polar [19,20].

The magnetic field in living cells may generate membrane depolarization of the target cell, which will allow calcium (Ca^2+^) ions to be released from the sarcoplasmic reticulum, thereby increasing Ca^2+^ levels in the target cell [21,22]. An increase in Ca^2+^ ion levels causes an increase in GLUT4, which appears on the surface of the target cell. The increased GLUT4 will lead to an increase in glucose from the blood to peripheral tissue, which will result in a decrease in blood glucose levels [23].

The electric field on the surface of the body will propel the stimulation of nerve receptors over the body’s entire surface area and cause an electric induction current that will affect the body’s cell ­system. Applying the electric field will be followed by the movement of body hair, indicating that stimulation of the electric field has been received by the body. Furthermore, this electrical signal will be transmitted to the brain, causing stimulation to specific parts thereof and will affect the hormonal system and the autonomic nervous system. The electric field can induce changes in transmembrane potential, leading to biochemical and physiological changes in cells, which may result in the vasodilation of blood vessels, enhancing blood viscosity, as well as a decrease in blood viscosity [23].

Infrared light plays an important role in the growth of living things. Living organisms on earth comprise water molecules and complex proteins; however, water molecules are not always stable and if they are oxidized at a wavelength of 8 to 1 μm, resonance will occur. Resonance causes ionized water become hydrogen and hydroxyl ions at very high speed (10/12 s). If ionization (also known as the activation of water) occurs in the body, the metabolic processes and the disposal of metabolic waste will become more active and effective, thus affecting a good impact on blood sugar. The dielectric properties of tissue will depend on the water content, which is an important factor in the interaction of electrical field with living things [24].

The administration of red-to-infrared light will produce a biomodulation effect [25] if the process occurs in the body and improves the blood sugar level through a resonance process, resulting in ionization through water activation [10]. Thus, the metabolism process and the disposal of metabolic waste become more active and more effective [11,12]. Furthermore, red and infrared light increases the blood flow rate because it also has a vasodilation effect [6,10] and increases body temperature. Hyperthermia will increase the concentration of free Ca^2+^ in the cytosol, which can eventually induce GLUT4 translocation in striated muscle cells, thereby causing glucose uptake from the blood to increase [6,13].

Infrared is a type of electromagnetic radiation that has a wavelength between visible light and radio waves [26]. This study used an NIR light with a wavelength of 941 nm and an intensity of 4 mW/cm^2^, generated by an LED with 1 mW output at the 20 cm range. It is known that the body contains electrolyte fluid, that is, a solution of ions (positively and negatively electrically charged) in which the vibration frequency is the same as the infrared frequency; therefore, if the body is exposed to infrared rays, it will absorb photons/infrared energy, resulting in electrons being excited. Then, a transition to the ground state will occur through the emitting of biophotons, which generate heat and will increase body temperature or give rise to hyperthermia. Hyperthermic conditions will increase the concentration of free Ca2 in the cytosol, which can eventually induce translocation of GLUT4 in striated muscle cells, which will increase glucose uptake from the blood [5]. This can increase the blood flow rate as a result of vasodilation effects [10,11]. Red/NR light stimulates the release of an endothelium-dependent vasodilator and helps to avoid vascular dysfunction [27]. Therefore, the increase in temperature in the red and infrared spectrum can be used for additional treatment in medicine with a relatively fast healing time [12].

## Conclusion

This study results showed that combined PS treatment for DC treatment group caused changes in blood sugar levels that were not significantly different from the NC group. The PS treatment’s improvement of Langerhans islet diameter was also not significantly different compared with the NC group. Accordingly, the combined PS treatment is effective for reducing blood sugar levels in diabetic mice and improving the diameter of Langerhans islets.

## Authors’ Contributions

SS, SDA, and APP collected the materials, performed the experiment, analyzed the data, and wrote the manuscript. SAH, DW and DAR prepared and analyzed the organ histology, while ATM and MM participated in analyzing animal experiments, correction, and proofread the manuscript. All authors read and approved the final manuscript.
